# Association between neutrophil to high-density lipoprotein cholesterol ratio and 28-day mortality in sepsis patients: Analysis from the MIMIC-IV database

**DOI:** 10.1097/MD.0000000000046397

**Published:** 2025-12-12

**Authors:** Li-Hua Hang, Jing Zhou, Zilian Luo, Li Zhang

**Affiliations:** a Department of Anesthesiology, Kunshan First People’s Hospital Affiliated to Jiangsu University, Kunshan, Jiangsu, China; b Kunshan Cancer Pain Prevention and Treatment Key Laboratory, Kunshan, Jiangsu, China.

**Keywords:** high-density lipoprotein cholesterol, mortality, neutrophil counts, neutrophil-to-high-density lipoprotein cholesterol ratio, sepsis

## Abstract

The neutrophil count-to-high-density lipoprotein cholesterol ratio (NHR), a composite biomarker of lipid metabolism and inflammation, has been regarded as an indicator for predicting clinical outcomes in patients suffering from acute cardiovascular and cerebrovascular disorders. However, the association between the NHR and 28-day all-cause mortality in the sepsis population remains unclear. Our study population comprised 1018 sepsis patients in the Medical Information Mart for Intensive Care IV (MIMIC-IV V2.2) database who were grouped into tertiles based on their NHRs. In this study, curve fitting and 2-piecewise regression models were utilized to analyze the dose–response relationship between the Log NHR and 28-day mortality. The mean age of the participants in the study cohort was 65.0 ± 15.7 years, and 56.3% were men. The 28-day mortality rates for the NHR tertiles were 15% (51), 13.3% (45), and 18.2% (62). By investigating the Log NHR and 28-day mortality, we observed a clear U-shaped relationship. Threshold saturation analysis revealed that when Log NHR below 1.87 (NHR 6.50), with each 1-unit increase in the Log NHR, the risk of 28-day mortality significantly decreased (HR = 0.61, 95% confidence interval: 0.37, 1.01, *P* = .0526). Conversely, the logNHR for 28-day mortality increased beyond 1.87 (HR = 1.72, 95% confidence interval: 1.18, 2.51; *P* = .0045). Stratified by age, our analysis revealed nuanced variations in the Log NHR and 28-day mortality association across different age groups. This study revealed a robust U-shaped relationship between the Log NHR and 28-day all-cause mortality in the sepsis population, with a critical threshold of Log NHR = 1.87 (NHR 6.50). This risk is influenced by age.

## 1. Introduction

Sepsis is a potentially fatal organ dysfunction caused by a dysregulated host response to infection. The incidence of sepsis has been increasing globally, with estimates suggesting that there could be 48 million cases annually worldwide.^[1]^ The yearly global incidence of sepsis is approximately 30 million, resulting in 6 million deaths.^[2]^ Delayed diagnosis, late treatment, and nonadherence to treatment guidelines contribute to higher mortality rates.^[3]^

Research indicates that during sepsis, neutrophils undergo impaired migration to the infection site and altered antimicrobial activity, leading to dysfunction and potential organ failure.^[4]^ In sepsis, neutrophil activation triggers the release of proinflammatory cytokines, reactive oxygen species, and neutrophil extracellular traps, which can exacerbate tissue damage and perpetuate systemic inflammation.^[5]^ Excessive neutrophilia often reflects a hyperinflammatory state that correlates with disease severity and poor outcomes in septic patients.^[6]^

Clinical data indicate a rapid decrease in high-density lipoprotein (HDL) cholesterol levels during sepsis, with lower levels correlating with adverse outcomes.^[7]^ HDL cholesterol plays a protective role in sepsis through various mechanisms.^[7]^ HDL is believed to promote hormone synthesis, clear bacterial toxins, protect the endothelial barrier, and exert antioxidant, anti-inflammatory, and anti-infective effects.^[7]^ Injection of HDL in sepsis animal models has been demonstrated to improve survival rates, suggesting the potential application of HDL therapy in human sepsis^[7]^; this is evident in both observational epidemiology^[8]^ and randomized studies in genetics.^[9]^ Furthermore, HDL particles can directly neutralize bacterial lipopolysaccharide and inhibit neutrophil activation, thereby counteracting the neutrophil-mediated inflammatory cascade in sepsis.^[10]^

The neutrophil-to-high-density lipoprotein cholesterol ratio (NHR) has been identified as a significant predictor of mortality across diverse patient populations. Multiple studies have confirmed the prognostic value of the NHR in various contexts. For example, research by Ozgeyik et al indicated that the NHR is a superior long-term predictor of mortality compared to other lipid or cell-related biomarkers.^[11]^ Moreover, an increased NHR has emerged as a strong independent predictor of cardiovascular and all-cause mortality.^[12]^ In addition, for patients with hepatocellular carcinoma,^[13]^ the NHR demonstrated an AUC of 0.740. For predicting acute coronary syndrome in patients with type 2 diabetes mellitus,^[14]^ an NHR ≥4.32 had an AUC of 0.722, highlighting its potential as a clinical indicator for predicting outcomes in different patient groups. The 2 haematological parameters are inexpensive, well standardized, and easy to measure. These characteristics make the NHR a promising clinical indicator.

Based on a thorough literature review, prior research has preliminarily discussed the prognostic relevance of the NHR in other diseases.^[13,15]^ However, specific associations with sepsis remain relatively limited. The aim of this study was to address this knowledge gap by comprehensively collecting clinical data and conducting in-depth analyses of the correlation between the NHR and 28-day mortality in sepsis patients, providing more accurate predictive indicators for the clinical management of sepsis patients.

## 2. Materials and methods

### 2.1. Study design

The current work adhered to the standards outlined in the STROBE statement. This retrospective investigation of patients with sepsis was longitudinal and single-center.

### 2.2. Patient and public involvement

The study did not include active participation from patients or the general public.

### 2.3. Data source

The Medical Information Mart for Intensive Care IV (MIMIC-IV V2.2) database is a carefully curated and identifiable collection of medical records from patients hospitalized in the intensive care unit (ICU) between 2008 and 2019. The Ethics Committee of Kunshan First People’s Hospital approved the study (Ethics Number 2024-04-001-k01).

### 2.4. Study population

**Figure 1.**
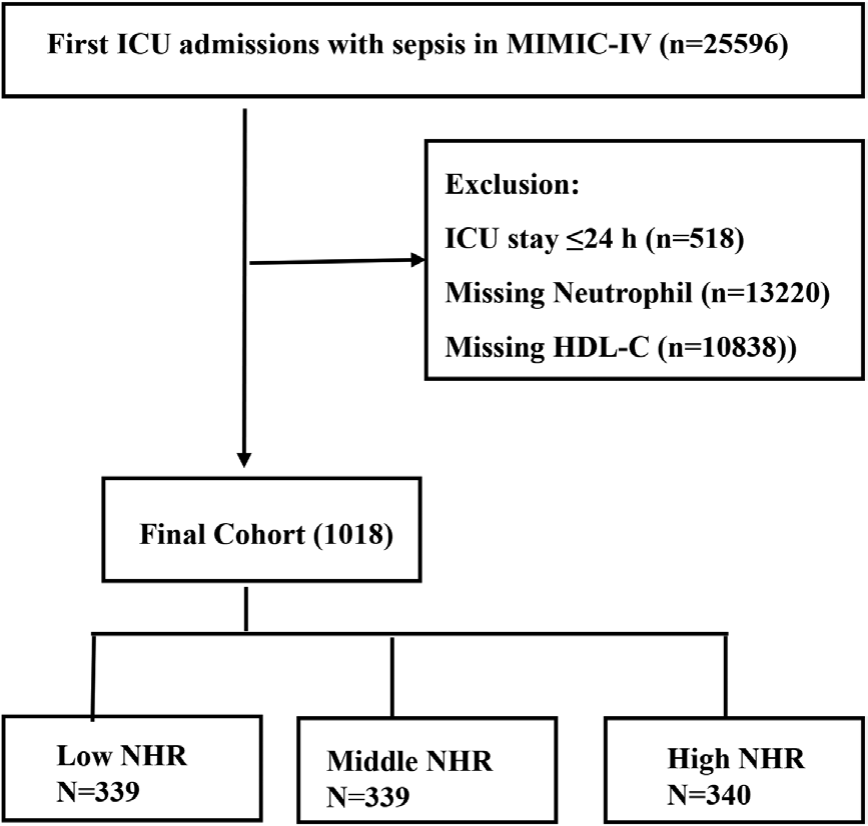
Flowchart of patient selection. Exclusion and inclusion criteria for selecting the final cohort of 1018 patients.

The study included 1018 individuals who were diagnosed with sepsis and whose BMI data were available. Participants were recruited from the MIMIC-IV database.^[16]^ The Third International Consensus Definitions for Sepsis and Septic Shock (Sepsis-3) guidelines were used to describe sepsis. The initial screening included patients with a sepsis diagnosis, including those with sepsis, severe sepsis, and septic shock (ICD9 codes: 99591, 99592, and 78552, respectively). The main inclusion criterion was sepsis in adult patients (aged 18–89 years). The major exclusion criteria were as follows: a length of ICU stay <24 hours, non-first admission to the ICU, and missing neutrophil or HDL cholesterol data at ICU admission as shown in Figure 1.

### 2.5. Data retrieval

The data were extracted with Structured Query Language. Patient age, sex, height, weight and Charlson comorbidity index score were recorded. Records about the administration of vasopressors, mechanical ventilation, and sedatives within the first 24 hours after admission to the ICU were also collected. Comorbidity data, such as diabetes mellitus, congestive heart failure, coronary artery disease (CAD), hypertension, atrial fibrillation, stroke, renal disease, liver disease, chronic pulmonary disease, and malignant tumors, were gathered using the International Classification of Diseases coding systems. The initial data collected at the onset of sepsis included vital signs (heart rate and minimum arterial pressure), severity of illness (simplified acute physiologic score [SAPS], sequential organ failure assessment [SOFA], Charlson comorbidity index), laboratory tests (partial pressure of oxygen [PO_2_], hemoglobin concentration, white blood cell count, absolute peripheral platelet counts, neutrophil counts, monocyte counts, lymphocyte counts, HDL cholesterol, lactate, creatinine, and pH levels).

### 2.6. Exposure and outcomes

The NHR was calculated from the neutrophil count (N, ×10^9^/L) and high-density lipoprotein cholesterol (H, HDL-C, mmol/L) using the following formula: NHR = N/H. The values were also transformed to a logarithmic scale to minimize the skewness of the underlying distribution. This study presents a model of the tertiles of the exposure distribution to assess the potential association between the NHR and the outcomes. The primary outcome assessed was mortality due to any cause within 28 days. The secondary outcome examined was the duration of stay in the ICU.

### 2.7. Statistical analysis

This retrospective study did not include an a priori statistical analysis strategy or statistical power calculations. Based on the data that were already present in the database, the sample size was selected. All missing values, entries with recording errors, and other potential confounding factors with missing values exceeding 10% were excluded. Table S1, Supplemental Digital Content, https://links.lww.com/MD/Q870 shows the variable missing rates. Missing values for each variable were estimated using multiple imputations.^[17]^ Multicollinearity among variables was detected using the variance inflation factor. The absence of multicollinearity for each variable was indicated by a variance inflation factor <5 (Table S2, Supplemental Digital Content, https://links.lww.com/MD/Q870).

The NLR was categorized into 3 tertiles. The patients were grouped according to age into 3 categories: <65, 65 to 80, and ≥80 years. Baseline characteristics were described in accordance with the tertiles of NHRs (<5.37, 5.37–10.57, and >10.57). Categorical variables are represented using numbers and percentages, and between-group differences were assessed using chi-squared and Fisher’s exact tests. Continuous variables are presented as medians and interquartile ranges, and between-group differences were analyzed using the Mann–Whitney *U* test. A univariate Cox regression model was applied to assess the associations between factors of interest and 28-day all-cause mortality (Table S3, Supplemental Digital Content, https://links.lww.com/MD/Q870). For covariate screening, the study employed LASSO (Least Absolute Shrinkage and Selection Operator) regression to identify relevant covariates (Table S4, Supplemental Digital Content, https://links.lww.com/MD/Q870). Variables with non-zero coefficients in the LASSO model and *P* < .10 were selected as adjustment variables for the multivariate analysis, with additional consideration given to their clinical relevance in sepsis management. This approach resulted in the selection of 9 covariates: age, SAPS II score, SOFA score, Charlson comorbidity index, ventilation status, vasopressor use, diabetes status, CAD status, and stroke status. Due to the nonnormal distribution of NHR data, logarithmic transformation was applied to derive the Log NHR. A generalized additive model was utilized for detecting nonlinear relationships. Upon identification of a nonlinear relationship, a 2-piecewise linear regression model was constructed,^[18]^ employing the obtained smoothing plot to quantify the threshold impact of the Log NHR on the 28-day mortality rate.

Several sensitivity analyses were conducted, including age-stratified analysis; categorizing septic patients with comorbidities such as atrial fibrillation, heart failure, CAD, stroke, or hypertension as having cardiovascular and cerebrovascular diseases for analysis; and excluding the influence of extreme NLRs on 28-day mortality.

The statistical analyses were conducted using R software (version 4.2.3; R Foundation for Statistical Computing, Vienna, Austria). Statistical significance was determined at a 2-tailed *P* value of <.05.

## 3. Results

The detailed patient selection process is illustrated in Figure 1. Among a total of 25,596 patients meeting the diagnostic criteria for sepsis, 1018 fulfilled the inclusion criteria for the study.

The baseline characteristics of the 1018 inpatients with sepsis are shown in Table 1. Stratified into tertiles, participants with low (NHR < 5.37), middle (5.37 < NHR < 10.57), and high (NHR > 10.57) levels displayed distinctive demographic and clinical features. Remarkably, there was considerable variation in age distribution (*P* < .001), with participants having an average age of 65.0 ± 15.7 years. The high-NHR group had a lower average age. The sex distribution also differed, with a greater proportion of males in the high-NHR category (*P* < .001). Clinical scores, including the SAPS, SOFA, and Charlson comorbidity index, increased with increasing NHR tertiles (*P* < .001, .003, .004, respectively), indicating potential associations with disease severity. Moreover, interventions such as mechanical ventilation and vasopressor use were significantly different across the NHR groups (*P* = .051 and <.001, respectively). Various comorbidities (including atrial fibrillation, hypertension, renal disease, liver disease, chronic obstructive pulmonary disease, CAD, and stroke), vital signs (heart rate, mean arterial pressure), and laboratory test results (hemoglobin, monocyte count, neutrophil count, WBC max count, platelet count, lymphocyte count, HDL-C, pH min, PO_2_ min, creatinine max, and lactate max) exhibited noteworthy distinctions. Transformed variables (monocyte to high-density lipoprotein cholesterol ratio, platelet to high-density lipoprotein cholesterol ratio, lymphocyte to high-density lipoprotein cholesterol ratio, and Log NHR) also demonstrated significant differences.

**Table 1:** Baseline characteristics of participants by tertiles of the neutrophil to high-density lipoprotein cholesterol ratio (NHR).

Variables	Total (n = 1018)	Low (NHR < 5.37) n = 339	Middle (5.37 < NHR < 10.57) n = 339	High (NHR > 10.57) n = 340	*P* value
Age (yr)	65.0 ± 15.7	66.6 ± 15.2	66.2 ± 16.1	62.2 ± 15.6	<.001
Age, n (%)					.002
<60	483 (47.4)	156 (46)	142 (41.9)	185 (54.4)	
60–80	326 (32.0)	102 (30.1)	117 (34.5)	107 (31.5)	
>80	209 (20.5)	81 (23.9)	80 (23.6)	48 (14.1)	
Gender, n (%)					<.001
Female	445 (43.7)	172 (50.7)	149 (44)	124 (36.5)	
Male	573 (56.3)	167 (49.3)	190 (56)	216 (63.5)	
Weight (kg)	83.2 ± 24.8	79.2 ± 23.9	82.5 ± 23.9	87.8 ± 25.9	<.001
Height (cm)	168.4 ± 14.9	167.3 ± 15.0	167.8 ± 15.5	170.0 ± 14.2	.122
SAPS score, median (IQR)	36.5 (29.0, 45.0)	35.0 (28.0, 42.0)	36.0 (29.0, 44.0)	39.0 (30.0, 48.2)	<.001
SOFA, median (IQR)	1.0 (0.0, 3.0)	1.0 (0.0, 3.0)	1.0 (0.0, 3.0)	2.0 (0.0, 4.0)	.003
Charlson comorbidity index, (IQR)	6.0 (4.0, 8.0)	6.0 (4.0, 8.0)	6.0 (4.0, 8.0)	5.0 (3.0, 7.0)	.004
Interventions					
Mechanical ventilation use, n (%)					.051
No	172 (16.9)	71 (20.9)	50 (14.7)	51 (15)	
Yes	846 (83.1)	268 (79.1)	289 (85.3)	289 (85)	
Vasopressor use, n (%)					<.001
No	623 (61.2)	236 (69.6)	220 (64.9)	167 (49.1)	
Yes	395 (38.8)	103 (30.4)	119 (35.1)	173 (50.9)	
Sedative use, n (%)					.966
No	922 (90.6)	308 (90.9)	306 (90.3)	308 (90.6)	
Yes	96 (9.4)	31 (9.1)	33 (9.7)	32 (9.4)	
Comorbidity					
CHF, n (%)					.262
No	957 (94.0)	314 (92.6)	318 (93.8)	325 (95.6)	
Yes	61 (6.0)	25 (7.4)	21 (6.2)	15 (4.4)	
AFIB, n (%)					.014
No	692 (68.0)	251 (74)	220 (64.9)	221 (65)	
Yes	326 (32.0)	88 (26)	119 (35.1)	119 (35)	
Diabetes, n (%)					.42
No	687 (67.5)	238 (70.2)	225 (66.4)	224 (65.9)	
Yes	331 (32.5)	101 (29.8)	114 (33.6)	116 (34.1)	
Hypertension, n (%)					.028
No	600 (58.9)	181 (53.4)	204 (60.2)	215 (63.2)	
Yes	418 (41.1)	158 (46.6)	135 (39.8)	125 (36.8)	
Renal disease, n (%)					.001
No	179 (17.6)	80 (23.6)	54 (15.9)	45 (13.2)	
Yes	839 (82.4)	259 (76.4)	285 (84.1)	295 (86.8)	
Liver disease, n (%)					.045
No	784 (77.0)	258 (76.1)	276 (81.4)	250 (73.5)	
Yes	234 (23.0)	81 (23.9)	63 (18.6)	90 (26.5)	
COPD, n (%)					.002
No	922 (90.6)	322 (95)	296 (87.3)	304 (89.4)	
Yes	96 (9.4)	17 (5)	43 (12.7)	36 (10.6)	
CAD, n (%)					.002
No	820 (80.6)	294 (86.7)	266 (78.5)	260 (76.5)	
Yes	198 (19.4)	45 (13.3)	73 (21.5)	80 (23.5)	
Stroke, n (%)					<.001
No	563 (55.3)	177 (52.2)	170 (50.1)	216 (63.5)	
Yes	455 (44.7)	162 (47.8)	169 (49.9)	124 (36.5)	
Metastatic cancer, n (%)					.073
No	827 (81.2)	262 (77.3)	283 (83.5)	282 (82.9)	
Yes	191 (18.8)	77 (22.7)	56 (16.5)	58 (17.1)	
Vital signs					
Heart rate min (bpm)	60.5 ± 13.6	59.7 ± 13.5	60.5 ± 13.7	61.3 ± 13.6	.309
Heart rate max (bpm)	121.1 ± 26.0	117.2 ± 25.8	119.3 ± 22.7	126.6 ± 28.4	<.001
MAP (mm Hg)	55.4 ± 12.5	57.4 ± 12.6	55.9 ± 12.1	52.7 ± 12.3	<.001
Laboratory tests					
Hemoglobin (g/dL)	8.9 ± 2.2	9.3 ± 2.3	9.1 ± 2.2	8.3 ± 2.1	<.001
pH (IQR)	7.3 (7.2, 7.4)	7.3 (7.3, 7.4)	7.3 (7.3, 7.4)	7.3 (7.2, 7.4)	<.001
PO_2_ (IQR)	48.0 (35.0, 70.0)	56.0 (39.0, 74.5)	50.5 (35.2, 74.0)	41.0 (33.0, 61.0)	<.001
Creatinine (IQR)	1.1 (0.8, 1.7)	1.0 (0.7, 1.4)	1.1 (0.8, 1.6)	1.3 (0.9, 2.3)	<.001
Lactate (IQR)	2.3 (1.5, 3.9)	2.2 (1.5, 3.6)	2.1 (1.4, 3.2)	2.9 (1.7, 5.1)	<.001
WBC count (10^9^/L), median (IQR)	15.6 (11.5, 20.6)	12.4 (9.5, 16.6)	14.8 (11.1, 19.1)	19.4 (15.8, 26.5)	<.001
Monocyte count (10^9^/L), median (IQR)	0.7 (0.5, 1.0)	0.6 (0.4, 0.7)	0.7 (0.5, 0.9)	0.9 (0.6, 1.3)	<.001
Neutrophil count (10^9^/L), median (IQR)	7.5 (4.7, 11.4)	4.2 (3.0, 5.7)	7.7 (5.8, 9.5)	12.7 (9.4, 16.2)	<.001
Lymphocyte count (10^9^/L), median (IQR)	1.2 (0.8, 1.8)	1.3 (0.9, 1.9)	1.2 (0.8, 1.9)	1.1 (0.7, 1.6)	<.001
Platelet count (10^9^/L), median (IQR)	190.0 (138.0, 249.8)	176.0 (126.0, 227.0)	200.0 (148.0, 255.5)	192.5 (140.0, 264.0)	<.001
HDL-C (mmol), median (IQR)	1.0 (0.7, 1.3)	1.3 (1.0, 1.6)	1.0 (0.8, 1.3)	0.7 (0.5, 0.9)	<.001
NHR, median (IQR)	7.4 (4.5, 12.8)	3.5 (2.6, 4.5)	7.4 (6.3, 8.8)	16.3 (12.8, 24.5)	<.001
MHR, median (IQR)	0.7 (0.4, 1.1)	0.4 (0.3, 0.6)	0.7 (0.5, 1.0)	1.3 (0.8, 2.0)	<.001
PHR, median (IQR)	182.7 (124.3, 282.5)	132.3 (90.3, 181.0)	190.0 (128.5, 274.2)	278.5 (178.7, 428.6)	<.001
LHR, median (IQR)	1.2 (0.7, 2.0)	1.1 (0.7, 1.6)	1.2 (0.7, 2.0)	1.5 (0.9, 2.8)	<.001
Log NHR, median (IQR)	2.0 (1.5, 2.6)	1.2 (0.9, 1.5)	2.0 (1.8, 2.2)	2.8 (2.6, 3.2)	<.001

Data were expressed as mean ± SD/median (interquartile ranges [IQR]) for continuous variables and percentage for categorical variables.

AFIB = atrial fibrillation, CAD = coronary artery disease, CHF = congestive heart failure, COPD = chronic obstructive pulmonary disease, IQR = interquartile range, LHR = lymphocyte to high-density lipoprotein cholesterol ratio, MAP = mean arterial pressure, MHR = monocyte to high-density lipoprotein cholesterol ratio, NHR = neutrophil to high-density lipoprotein cholesterol ratio, PHR = platelet to high-density lipoprotein cholesterol ratio, SAPS = simplified acute physiology score, SOFA = sequential organ failure assessment.

**Figure 2.**
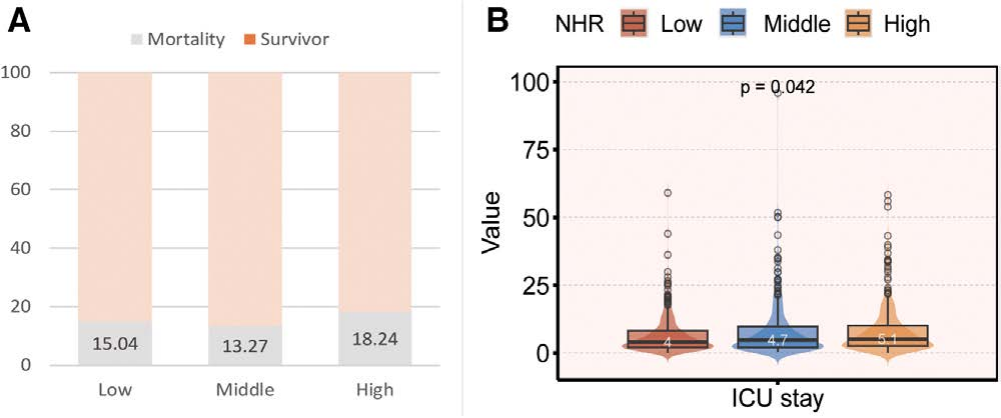
NHR 3-tier mortality rates (A) and median ICU admission duration (B). ICU = intensive care unit, NHR = neutrophil to high-density lipoprotein cholesterol ratio.

The low-NHR group had 84.96% survival, the middle-NHR group had 13.27% mortality, and the high-NHR group had 18.24% mortality (*P* = .195; Fig. 2A). The median ICU stay differed among the groups, with values of 4.0 days (interquartile range [IQR]: 2.1–8.2), 4.7 days (IQR: 2.1–9.8), and 5.1 days (IQR: 2.6–10.1) for the low, middle, and high groups, respectively (*P* = .042; Fig. 2B).

Table S3, Supplemental Digital Content, https://links.lww.com/MD/Q870 shows the univariate analysis for each variable in relation to 28-day mortality. Age, weight, SAPS score, Charlson comorbidity index, heart failure, atrial fibrillation, CAD, diabetes, kidney disease, liver disease, stroke, white blood cell count, neutrophil count, duration of mechanical ventilation, and use of vasoactive drugs are associated with the 28-day mortality rate. This study utilized LASSO regression to select 9 covariates, including age, SAPS score, SOFA score, Charlson comorbidity index score, CAD status, diabetes status, stroke status, mechanical ventilation status, and vasoactive drug usage, as adjustment variables (Table S4, Supplemental Digital Content, https://links.lww.com/MD/Q870).

**Figure 3.**
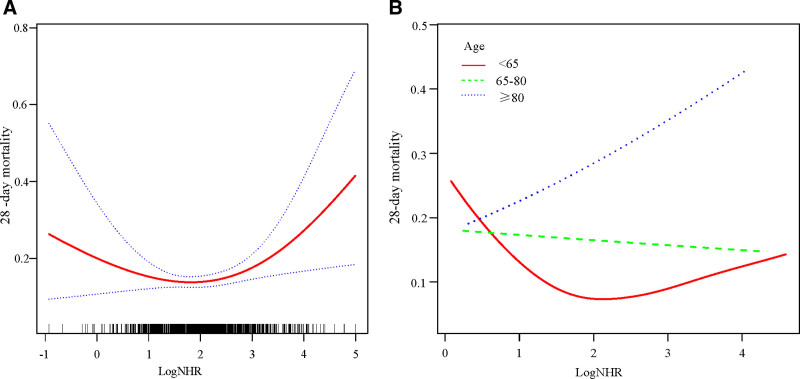
Fitted curves depicting the association between the Log NHR and 28-day mortality in the overall cohort (A) and across different age groups (B). The analysis was adjusted for age, simplified acute physiology score, sequential organ failure assessment, Charlson comorbidity index, mechanical ventilation use, vasopressor use, diabetes, coronary artery disease, and stroke. The solid lines represent the estimated 28-d mortality rates, while the dashed lines represent the corresponding 95% confidence intervals. NHR = neutrophil to high-density lipoprotein cholesterol ratio.

After adjusting for covariates, the relationship between the log-transformed NHRs and 28-day mortality exhibited a U-shaped nonlinear pattern (Fig. 3A). The 28-day mortality significantly varied based on Log NHR values, being below or above 1.87. Particularly, with a Log NHR below 1.87, there was a 39% decrease in the 28-day mortality risk for every 1-unit decrease (HR 0.61, 95% confidence interval [CI]: 0.37–1.01; *P* = .0526). In contrast, when the Log NHR exceeded 1.87, the risk of 28-day mortality increased by 72% for every 1-unit increase (HR 1.72, 95% CI: 1.18–2.51; *P* = .0045; Table 2). For cardiovascular and cerebrovascular diseases, when the Log NHR was below 1.81, there was a 38% reduction in the risk of 28-day mortality for every 1-unit decrease (HR 0.62, 95% CI: 0.39–1.00; *P* = .0485). Conversely, when the Log NHR exceeded 1.81, there was a 61% increase in the risk of 28-day mortality for every 1-unit increase (HR 1.61, 95% CI: 1.15–2.25; *P* = .0053; Table S5 and Fig. S1, Supplemental Digital Content, https://links.lww.com/MD/Q870). Sensitivity analysis for the impact of the Log NHR on 28-day mortality, after excluding outliers, showed results similar to those of the main analysis (Table S6 and Fig. S2, Supplemental Digital Content, https://links.lww.com/MD/Q870). The relationship between the Log NHR and 28-day mortality across different age groups is shown in Figure 3B. In the age group younger than 65 years, there is a U-shaped correlation between the 2 variables. In the age range of 65 to 80 years, the association appears relatively flat. Among patients aged 80 years and above, there was a significant increase in the risk of 28-day mortality with the Log NHR.

**Table 2:** The result of the 2-piecewise linear regression model.

	28-d mortality (HR, 95% CI, *P*)
Fitting model by standard linear regression	1.15 (0.90, 1.46) .2608
Fitting model by 2-piecewise linear regression	
Inflection point of Log NHR	1.87 (NHR 6.50)
<1.87	0.61 (0.37, 1.01) .0526
>1.87	1.72 (1.18, 2.51) .0045
*P* for log-likelihood ratio test	.007

We adjusted age, simplified acute physiology score, sequential organ failure assessment, charlson comorbidity index, mechanical ventilation use, vasopressor use, diabetes, coronary artery disease, stroke.

CI = confidence interval, HR = hazard ratio, NHR = neutrophil to high-density lipoprotein cholesterol ratio.

## 4. Discussion

The aim of this study was to assess the relationship between 28-day mortality and NHRs in sepsis inpatients using the MIMIC-IV database. The analysis revealed a dose–response relationship between the Log NHR and 28-day mortality, revealing a distinctive curvilinear pattern indicative of a threshold effect. Specifically, Log NHR levels below 1.87 (NHR 6.50) were associated with a reduction in 28-day mortality, while levels above 1.87 were linked to an increase in 28-day mortality. Notably, the nature of this relationship varied across different age groups.

Recently, a growing body of research has been dedicated to exploring potential risk markers for clinical outcomes, particularly focusing on composite predictors involving haematological parameters. These markers, which originate from routine blood tests, are affordable and accessible. Compared to individual parameters, the ratios of various parameters stand out for their capacity to provide comprehensive information and exhibit comparable predictive abilities. Li et al explored the efficiency of the monocyte/high-density lipoprotein cholesterol ratio in predicting 28-day mortality in sepsis patients, adding to the growing body of literature on lipid-related prognostic markers.^[19]^ The NHR excels in capturing the complexity of pathological processes associated with both inflammatory reactions and abnormal lipid metabolism, contributing to more accurate prognostication of outcomes. Several studies have demonstrated the prognostic value of the NHR in different contexts. A study by Shi et al investigated the NHR and its relevance to hepatocellular carcinoma mortality. Their findings suggest that an elevated NHR (≥3.5) is a powerful independent risk factor associated with a high mortality rate.^[13]^ One study indicated that the NHR is a better predictor of long-term mortality than any other lipid or cell-related biomarker in patients with Creutzfeldt–Jakob disease.^[20]^ The NHR has been correlated with disease severity in type 2 diabetic patients with peripheral arterial disease.^[21]^ The NHR has been proposed as a novel indicator for assessing the impact of inflammation and lipid abnormalities on CVD prognosis.^[12,22]^ In severe acute ischemic stroke patients, the NHR shows a notable increase and serves as an independent predictor of short-term outcomes.^[23]^ Another study suggested that the NHR can predict long-term mortality in elderly acute myocardial infarction patients.^[24]^ Additionally, in a retrospective cohort study, an increase in the NHR was considered an independent risk factor for all-cause mortality in peritoneal dialysis patients.^[25]^ However, the association between the NHR and 28-day all-cause mortality in the sepsis population remains unclear. Importantly, this study revealed a U-shaped relationship between NHRs and short-term mortality.

Sepsis is characterized by a dysfunctional immune response to infection, leading to damage to multiple organ systems.^[26]^ Inflammation is a necessary component for clearing infection, but the dysregulated inflammatory response in sepsis can lead to adverse outcomes.^[26]^ The neutrophil-to-lymphocyte ratio (NLR) has been studied in the context of sepsis severity and mortality prediction, but specific information about the NHR in sepsis patients is limited.^[27,28]^ While the NHR has been studied in the context of other health conditions, its specific role in sepsis has not been extensively documented. However, the relationships between lipid profiles and sepsis outcomes, as well as the importance of inflammation in sepsis, are well established. Further research may be needed to fully understand the implications of the NHR in sepsis.

Our study provided novel insight revealing a nonlinear association between the NHR and 28-day all-cause mortality. The underlying mechanism remains unclear, but 1 plausible explanation is the concentration of HDL-C among participants with a lower NHR. HDL cholesterol is thought to play a protective role in sepsis through several mechanisms. Clinical data demonstrate that HDL cholesterol levels decrease rapidly during sepsis, and low levels are correlated with poor outcomes.^[10]^ HDL is believed to promote steroid synthesis, clear bacterial toxins, protect the endothelial barrier, and exhibit antioxidant, anti-inflammatory, and anti-infectious functions.^[7,29]^ Additionally, HDL infusion in animal models of sepsis has been shown to improve survival, suggesting the potential of HDL therapy in human sepsis.^[7]^ A study by Bowe and colleagues revealed a U-shaped distribution of the risk of all-cause mortality associated with HDL-C levels, with a reduced risk observed only in individuals with intermediate HDL-C levels (between 25 and 50 mg/dL).^[30]^ In the hypertensive population, both lower and higher concentrations of HDL-C were linked to increased mortality.^[31]^ Huang et al emphasized the biphasic effect of HDL-C, where intermediate to high levels adversely affect endothelial progenitor cells and the associated vascular regeneration, leading to a loss of its protective role.^[32]^ In early septic shock, insufficient circulating neutrophils may compromise the effective innate immune response against invasive pathogens.^[33]^ Delayed neutrophil apoptosis and the release of immature cells contribute to increased circulating neutrophils, inducing cytotoxicity, an immune response, and tissue damage,^[34]^ as well as substantial production of the immunosuppressive cytokine IL-10.^[35]^ Activated neutrophils mediate HDL oxidation and impair cholesterol efflux through oxidant-generating enzymes such as MPO, NADPH oxidase, and nitric oxide synthase.^[12]^ In contrast, HDL-C exhibits anti-inflammatory effects by inhibiting neutrophil activation, adhesion, proliferation, and migration, which are associated with the abundance of lipid rafts.^[36]^

Our observations highlight that a lower NHR is linked to a greater average age, a greater proportion of females, and an increased occurrence of hypertension and heart failure. This finding implies that age, sex, hypertension, and heart failure might contribute to the increased mortality risk in this cohort. Additional studies are required to verify the impact of age and cancer on the correlation between a lower NHR and an increased risk of all-cause mortality. Conversely, an elevated NHR is correlated with a greater percentage of males, increased SAPS and SOFA scores, CAD, atrial fibrillation, liver and kidney diseases, diabetes, and increased use of vasoactive drugs; this suggests that male sex, disease severity scores, CAD, atrial fibrillation, liver and kidney diseases, diabetes, and increased use of vasoactive drugs contribute to the elevated risk of death in this cohort. Therefore, the correlation between a greater NHR and poorer prognosis can be explained by the involvement of neutrophils in atherosclerosis, inflammation, and cardiovascular events, as well as the impact of neutrophil interactions on HDL-C and cholesterol dynamics.

In this study, we utilized a threshold effect analysis to investigate the relationship between NHRs and 28-day mortality. The NHR exhibited a significant association with mortality, with the lowest mortality observed at an NHR of 6.50. Previous studies predominantly utilized ROC curves to predict mortality rates. For example, Ozgeyik et al reported a cutoff value of 0.269 for the NHR, achieving a sensitivity and specificity of 74.2% in predicting long-term mortality.^[11]^ Furthermore, distinct studies reported cutoff values of 1.51^[37]^ and 3.87 for the NHR for predicting significant coronary stenosis, while the cutoff values for predicting adverse cardiovascular outcomes and mortality were 5.74^[24]^ and 11.28,^[15]^ respectively. These findings suggest that the critical threshold of the NHR may vary across different outcome measures, which indicates the importance of considering the influence of various outcome indicators on the NHR threshold when assessing patients with sepsis for a more accurate evaluation of the severity and prognosis of the patients’ condition.

Age-stratified analysis revealed that the U-shaped relationship between NHR and mortality was more pronounced in younger patients (<65 years), while older patients (≥65 years) demonstrated a more linear relationship. We propose several explanations for this phenomenon.

Age-stratified analysis revealed that the U-shaped relationship between NHR and mortality was more pronounced in younger patients (<65 years), while older patients (≥65 years) demonstrated a more linear relationship. We propose several explanations for this phenomenon: Age-related immune senescence leads to decreased neutrophil function (reduced chemotaxis, phagocytosis) in older adults, potentially making neutrophil counts less reflective of actual immune function and thus affecting the prognostic value of NHR. With aging, HDL particles undergo structural and functional alterations, with reduced anti-inflammatory and reverse cholesterol transport capabilities, which may modify the relationship between HDL-C levels and their protective effects. Elderly patients typically have more chronic comorbidities and elevated baseline inflammation, which may mask the clinical significance of extreme NHR values, while in younger patients, NHR may more directly reflect acute pathological changes.

## 5. Limitations

Our investigation has notable strengths. First, we present a comprehensive body of evidence elucidating the prognostic significance of the NHR for 28-day all-cause mortality among sepsis patients. Second, for the first time, we introduce the conceptual association between the NHR and short-term outcomes by employing a curve-fitting model. However, the current study has certain limitations. Given its observational nature, establishing causality becomes challenging. Additionally, relying solely on a single baseline measurement, overlooking dynamic fluctuations, may not fully capture the trajectory of blood parameter variations. The generalizability of our findings to diverse populations is constrained by demographic differences among European and North American populations. Retrospective analyses are susceptible to information bias, potentially leading to inaccuracies and data incompleteness. Subsequent research endeavors should systematically address these limitations through prospective methodologies. Furthermore, despite meticulous adjustments for covariates, potential confounding effects persist due to unmeasured or nonincluded variables.

## 6. Conclusion

Our findings revealed a U-shaped relationship between the NHR and the risk of 28-day all-cause mortality in the sepsis population, with a critical threshold at an NHR = 1.87. This risk is influenced by age, an insight that emphasizes the pivotal role of this inflection point. Future research utilizing machine learning techniques, as demonstrated by a recent study,^[38]^ could further refine NHR’s prognostic value for more personalized risk assessment in diverse sepsis populations.

## Author contributions

**Formal analysis:** Li-Hua Hang, Jing Zhou, Li Zhang.

**Funding acquisition:** Li-Hua Hang.

**Writing – original draft:** Li Zhang.

**Writing – review & editing:** Li-Hua Hang, Zilian Luo.

## Supplementary Material
