# Supplementary material for: Association between neutrophil to high-density lipoprotein cholesterol ratio and 28-day mortality in sepsis patients: Analysis from the MIMIC-IV database
Source: Medicine (Baltimore). 2025 Dec 12;104(50):e46397. doi: 10.1097/MD.0000000000046397 (PMC12708204; doi:10.1097/MD.0000000000046397)

**Supplemental Table S1.** Missing data.

|  | Variable | Miss.freq | **Miss.percentage%** |
| --- | --- | --- | --- |
| **1** | Age | 0 | 0 |
| **2** | Gender | 0 | 0 |
| **4** | Height | 391 | 39.3104 |
| **5** | Weight | 12 | 1.1788 |
| **6** | SOFA | 1 | 0.0982 |
| **7** | SAPS score | 0 | 0 |
| **8** | Charlson comorbidity index | 0 | 0 |
| **9** | ICU stay | 0 | 0 |
| **10** | 28-day mortality | 860 | 84.4794 |
| **11** | Mechanical ventilation use | 0 | 0 |
| **12** | Vasopressor use | 0 | 0 |
| **13** | Sedative use | 0 | 0 |
| **14** | AFIB | 0 | 0 |
| **15** | CAD | 0 | 0 |
| **16** | CHF | 0 | 0 |
| **17** | COPD | 0 | 0 |
| **18** | Diabetes | 0 | 0 |
| **19** | Hypertension | 0 | 0 |
| **20** | Liverdisease | 0 | 0 |
| **21** | Metastatic cancer | 0 | 0 |
| **22** | Renaldisease | 0 | 0 |
| **23** | Stroke | 0 | 0 |
| **24** | Heart rate max | 0 | 0 |
| **25** | Heart rate min | 0 | 0 |
| **26** | MAP min | 0 | 0 |
| **27** | WBC max count | 0 | 0 |
| **28** | Lymphocyte count | 0 | 0 |
| **29** | Monocyte count | 0 | 0 |
| **30** | Neutrophil count | 0 | 0 |
| **31** | Platelet count | 0 | 0 |
| **32** | HDL-C | 0 | 0 |
| **33** | pH min | 216 | 21.2181 |
| **34** | PO2 min | 231 | 22.6916 |
| **35** | Lactate max | 236 | 23.1827 |
| **36** | Creatinine max | 7 | 0.6876 |
| **37** | Hemoglobin min | 0 | 0 |

**Supplemental Table S2.** Multicollinearity analysis.

| Term1 | coeff1 | Change.percentage1 | Term2 | coeff2 | Change.percentage2 | VIF | colinearity | select | select.VIF |
| --- | --- | --- | --- | --- | --- | --- | --- | --- | --- |
| Crude | 0.02 | Ref. | Full | 0.01 | Ref. | 1.325 | 0 | Ref. | Ref. |
| Age | 0.02 | 50.1 | age | 0.01 | -7 | 2.044 | 0 | Yes | Yes |
| Gender | 0.02 | 4.8 | gender1 | 0.01 | -1.6 | 1.82 | 0 | No | No |
| Weight | 0.02 | 18.6 | weight_first | 0.01 | 0.2 | 1.492 | 0 | Yes | Yes |
| Height | 0.01 | -21.3 | height | 0.02 | 30 | 1.91 | 0 | Yes | Yes |
| SAPS score | 0.01 | -38.1 | sapsii | 0.02 | 35 | 1.529 | 0 | Yes | Yes |
| SOFA | 0.02 | 3.4 | sofa | 0.01 | -0.7 | 1.432 | 0 | No | No |
| Charlson comorbidity index | 0.02 | 22.5 | charlson_comorbidity_index | 0.01 | -5 | 2.473 | 0 | Yes | Yes |
| Mechanical ventilation use | 0.02 | -6.1 | ventilation | 0.01 | 4.6 | 1.147 | 0 | No | No |
| Vasopressor use | 0.01 | -24.2 | vasopressors | 0.02 | 5.9 | 1.416 | 0 | Yes | Yes |
| Sedative use | 0.02 | 3.7 | sedative | 0.01 | 0.6 | 1.153 | 0 | No | No |
| CHF | 0.02 | -5.2 | CHF | 0.01 | 3.2 | 1.091 | 0 | No | No |
| AFIB | 0.02 | -1.3 | AFIB | 0.01 | -1.2 | 1.262 | 0 | No | No |
| Diabetes | 0.02 | -1.1 | diabetes | 0.01 | 5.6 | 1.45 | 0 | No | No |
| Renaldisease | 0.01 | -10 | renaldisease | 0.01 | 0.5 | 1.212 | 0 | Yes | Yes |
| Hypertension | 0.02 | -1.1 | hypertension | 0.01 | 1.5 | 1.23 | 0 | No | No |
| COPD | 0.02 | -1.4 | copd | 0.01 | 0.5 | 1.115 | 0 | No | No |
| Liverdisease | 0.02 | 13.9 | liverdisease | 0.01 | -2.8 | 1.364 | 0 | Yes | Yes |
| CAD | 0.02 | 0.2 | CAD | 0.01 | -6.7 | 1.333 | 0 | No | No |
| stroke | 0.02 | 29.4 | stroke | 0.01 | 4.1 | 1.591 | 0 | Yes | Yes |
| Metastatic cancer | 0.02 | 0.3 | malignancy | 0.01 | -2.3 | 1.227 | 0 | No | No |
| Platelet count | 0.02 | 0 | plt | 0.02 | 9.3 | 1.204 | 0 | No | No |
| Monocyte count | 0.02 | 7 | mon | 0.01 | -27.2 | 1.21 | 0 | Yes | Yes |
| Lymphocyte count | 0.02 | -4.7 | lym | 0.01 | 5.2 | 1.047 | 0 | No | No |

**Supplemental table S3.** Univariate Cox regression analysis.

| Item | HR (95%CI) | P (Wald's test) | P (LR-test) |
| --- | --- | --- | --- |
| Age (cont. var.) | 1.03 (1.02,1.05) | < 0.001 | < 0.001 |
| Gender: 1 vs 0 | 0.85 (0.62,1.16) | 0.311 | 0.312 |
| Weight (cont. var.) | 0.9902 (0.983,0.9974) | 0.008 | 0.006 |
| Height (cont. var.) | 1.0012 (0.9887,1.0139) | 0.846 | 0.845 |
| sapsii (cont. var.) | 1.04 (1.03,1.05) | < 0.001 | < 0.001 |
| SOFA (cont. var.) | 0.98 (0.92,1.05) | 0.645 | 0.642 |
| Charlson comorbidity index (cont. var.) | 1.15 (1.1,1.21) | < 0.001 | < 0.001 |
| Ventilation: 1 vs 0 | 1.86 (1.11,3.11) | 0.019 | 0.011 |
| Vasopressors: 1 vs 0 | 1.92 (1.4,2.63) | < 0.001 | < 0.001 |
| Sedative: 1 vs 0 | 0.69 (0.37,1.27) | 0.229 | 0.204 |
| CHF: 1 vs 0 | 0.29 (0.09,0.91) | 0.034 | 0.008 |
| AF: 1 vs 0 | 1.76 (1.29,2.41) | < 0.001 | < 0.001 |
| Diabetes: 1 vs 0 | 0.68 (0.48,0.97) | 0.035 | 0.03 |
| Hypertension: 1 vs 0 | 0.91 (0.66,1.25) | 0.559 | 0.558 |
| Renaldisease: 1 vs 0 | 1.82 (1.1,3.02) | 0.019 | 0.011 |
| Liverdisease: 1 vs 0 | 0.66 (0.43,1) | 0.048 | 0.039 |
| COPD: 1 vs 0 | 1.54 (0.97,2.44) | 0.066 | 0.081 |
| CAD: 1 vs 0 | 1.75 (1.24,2.47) | 0.002 | 0.002 |
| Stroke: 1 vs 0 | 2.66 (1.91,3.7) | < 0.001 | < 0.001 |
| Malignancy: 1 vs 0 | 1.2 (0.82,1.75) | 0.348 | 0.357 |
| Map min (cont. var.) | 0.97 (0.96,0.98) | < 0.001 | < 0.001 |
| Heart rate min (cont. var.) | 0.9947 (0.9832,1.0064) | 0.377 | 0.376 |
| Heart rate max (cont. var.) | 1.01 (1.01,1.02) | < 0.001 | < 0.001 |
| Creatinine max (cont. var.) | 1.03 (0.96,1.11) | 0.395 | 0.415 |
| pO2 min (cont. var.) | 0.9965 (0.9911,1.002) | 0.213 | 0.184 |
| Lactate max (cont. var.) | 1.1 (1.06,1.14) | < 0.001 | < 0.001 |
| Hemoglobin min (cont. var.) | 0.95 (0.89,1.02) | 0.171 | 0.167 |
| PH min (cont. var.) | 0.1 (0.03,0.4) | 0.001 | 0.002 |
| WBC max count (cont. var.) | 1.01 (1,1.02) | 0.004 | 0.017 |
| Neutrophil count (cont. var.) | 1.03 (1.01,1.05) | 0.01 | 0.019 |
| Monocyte count (cont. var.) | 0.97 (0.76,1.23) | 0.781 | 0.765 |
| Platelet count (cont. var.) | 1.0002 (0.9985,1.0018) | 0.848 | 0.848 |
| Lymphocyte count (cont. var.) | 0.86 (0.71,1.05) | 0.136 | 0.003 |
| HDL-C (cont. var.) | 1.09 (0.79,1.49) | 0.607 | 0.609 |
| LHR (cont. var.) | 0.95 (0.86,1.05) | 0.308 | 0.008 |
| NHR (cont. var.) | 1.01 (1.01,1.02) | 0.001 | 0.005 |

**Supplemental Table S4.** Covariate Screening for 28-Day Mortality with Lasso Regression Analysis.

| Variable | Estimate | HR | Std.Error | z.value | P.value |
| --- | --- | --- | --- | --- | --- |
| age | 0.012 | 1.013 | 0.008 | 1.595 | 0.111 |
| sapsii | 0.033 | 1.033 | 0.007 | 4.634 | 0 |
| sofa | -0.089 | 0.915 | 0.041 | -2.185 | 0.029 |
| charlson_comorbidity_index | 0.068 | 1.071 | 0.036 | 1.915 | 0.055 |
| ventilation | 0.471 | 1.602 | 0.272 | 1.731 | 0.083 |
| vasopressors | 0.581 | 1.788 | 0.19 | 3.066 | 0.002 |
| sedative | -0.437 | 0.646 | 0.325 | -1.346 | 0.178 |
| diabetes | -0.641 | 0.527 | 0.198 | -3.238 | 0.001 |
| CAD | 0.498 | 1.646 | 0.187 | 2.661 | 0.008 |
| stroke | 0.994 | 2.702 | 0.183 | 5.432 | 0 |
| NHR | 0.016 | 1.016 | 0.005 | 3.169 | 0.002 |
| LHR | -0.088 | 0.916 | 0.051 | -1.73 | 0.084 |

**Supplemental Table S5.** Sensitivity analysis. The result of the two-piecewise linear regression model in cardiovascular and cerebrovascular diseases

|  | 28-day mortality (HR, 95%CI, *p*) |
| --- | --- |
| Fitting model by standard linear regression | 1.14 (0.90, 1.43) 0.2827 |
| Fitting model by two-piecewise linear regression |  |
| Inflection point of NHR | 1.81 (NHR 6.14) |
| < 1.81 | 0.62 (0.39, 1.00) 0.0485 |
| > 1.81 | 1.61 (1.15, 2.25) 0.0053 |
| P for log-likelihood ratio test | 0.009 |

**Supplemental Table S6.** Sensitivity analysis.

|  | 28-day mortality (HR, 95%CI, *p*) |
| --- | --- |
| Fitting model by standard linear regression | 1.01 (0.81, 1.26) 0.9266 |
| Fitting model by two-piecewise linear regression |  |
| Inflection point of NHR | 1.71 (NHR 5.53) |
| < 1.71 | 0.53 (0.31, 0.91) 0.0205 |
| > 1.71 | 1.34 (0.99, 1.83) 0.0610 |
| P for log-likelihood ratio test | 0.015 |

**Supplemental Figure 1**. Fitted curves illustrating the correlation between LogNHR and 28-day mortality in cardiovascular and cerebrovascular diseases. We adjusted age, simpliﬁed acute physiology score, sequential organ failure assessment, charlson comorbidity index, mechanical ventilation use, vasopressor use, diabetes, coronary artery disease, stroke. Solid lines represent the estimated 28-day mortality rates, while dashed lines represent the corresponding 95% confidence intervals.


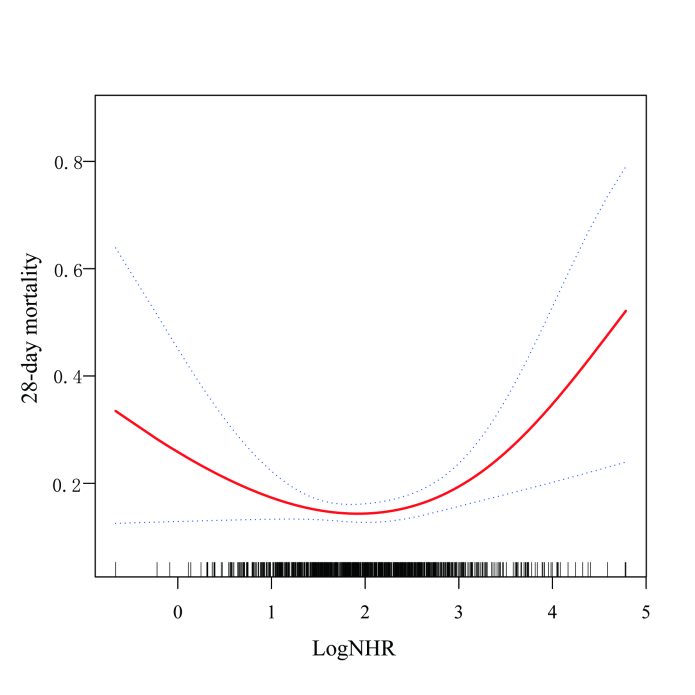


**Supplemental Figure 2**. Fitted curves illustrating the correlation between LogNHR and 28-day mortality after outlier removal. We adjusted age, simpliﬁed acute physiology score, sequential organ failure assessment, charlson comorbidity index, mechanical ventilation use, vasopressor use, diabetes, coronary artery disease, stroke. Solid lines represent the estimated 28-day mortality rates, while dashed lines represent the corresponding 95% confidence intervals.


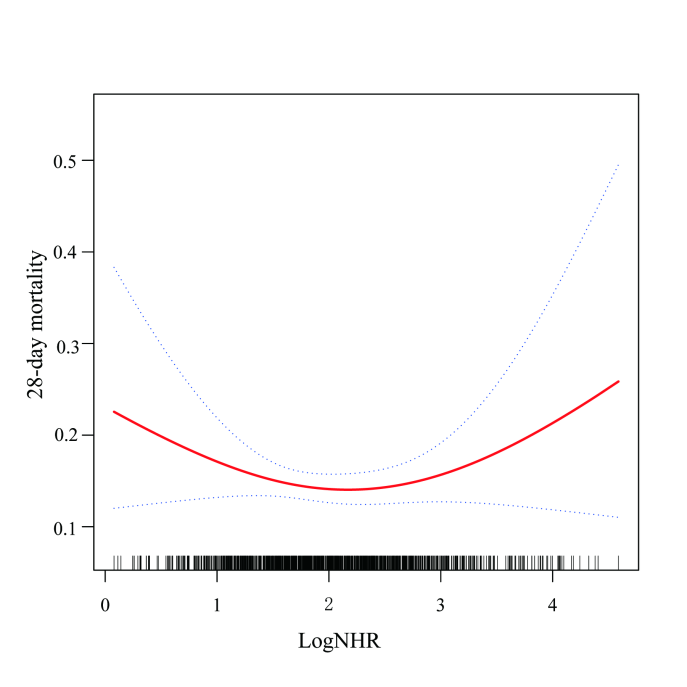

Supplement: Supplementary file 1 [file medi-104-e46397-s001.docx]
